# Expression and activation of the steroidogenic enzyme CYP11A1 is associated with IL-13 production in T cells from peanut allergic children

**DOI:** 10.1371/journal.pone.0233563

**Published:** 2020-06-04

**Authors:** Meiqin Wang, Matthew J. Strand, Bruce J. Lanser, Carah Santos, Kreso Bendelja, Jennifer Fish, Elizabeth A. Esterl, Shigeru Ashino, Jordan K. Abbott, Vijaya Knight, Erwin W. Gelfand

**Affiliations:** 1 Department of Pediatrics, Division of Cell Biology, National Jewish Health, Denver, CO, United States of America; 2 Division of Biostatistics and Bioinformatics, National Jewish Health, Denver, CO, United States of America; 3 Advanced Diagnostic Laboratories, National Jewish Health, Denver, CO, United States of America; University of Georgia, UNITED STATES

## Abstract

Activation of the steroidogenic enzyme CYP11A1 was shown to be necessary for the development of peanut-induced intestinal anaphylaxis and IL-13 production in allergic mice. We determined if levels of CYP11A1 in peripheral blood T cells from peanut-allergic (PA) children compared to non-allergic controls were increased and if levels correlated to IL-13 production and oral challenge outcomes to peanut. CYP11A1 mRNA and protein levels were significantly increased in activated CD4^+^ T cells from PA patients. In parallel, IL-13 production was significantly increased; IFNγ levels were not different between groups. There were significant correlations between expression levels of *CYP11A1* mRNA and levels of *IL13* mRNA and protein, levels of serum IgE anti-Ara h 2 and to outcomes of peanut challenge. The importance of CYP11A1 on cytokine production was tested using a *CYP11A1* CRISPR/Cas9 KO plasmid or an inhibitor of enzymatic CYP11A1 activity. Inhibition of CYP11A1 activation in patient cells treated with the inhibitor, aminoglutethimide, or CD4^+^ T cell line transfected with the CYP11A1 KO plasmid resulted in reduced IL-13 production. These data suggest that the CYP11A1-CD4^+^Tcell-IL-13 axis in activated CD4^+^ T cells from PA children is associated with development of PA reactions. CYP11A1 may represent a novel target for therapeutic intervention in PA children.

## Introduction

Peanut allergy is an important medical concern and often persists throughout life [1]. Peanut-induced anaphylaxis leads to social, psychological, and economic burdens [1, 2]. In recent important and paradigm-shifting studies, early feeding of peanut to high-risk infants resulted in significant decreases in the development of peanut allergy in children over the ensuing four years [3]. Thus, early exposure to peanut in a subset of non-sensitized patients offers a promising prevention strategy. For known or confirmed peanut-allergic (PA) patients, avoidance of peanut remains the only effective therapy and preventive measure to date, although new approaches are being explored in sensitized populations [4]. Although immunotherapy clinical trials for food allergy have been investigated for more than 10 years [5], no useful biomarkers are available for the diagnosis or prognosis of peanut allergy. Oral food challenge is the current gold-standard for the diagnosis of food allergies [6]. However, it has potential risks for severe allergic reactions including anaphylaxis [7]. Further, oral food challenge cannot be performed in non-specialized clinical centers as it is time-consuming, risky, and costly. Development of *in vitro* tests to assess susceptibility to food allergy, severity of an allergic reaction, or potential success of immunotherapy would be invaluable. This would require defining important biomarkers related to disease pathophysiology and correlations with clinical outcomes.

In a mouse model of peanut allergy, we identified increased expression and activation of a novel gene, cytochrome P450, family 11, subfamily A, polypeptide 1 (*CYP11A1*), to be a central regulator of IL-13 production and an essential component in the development of peanut-induced intestinal anaphylaxis [8]. CYP11A1 is the first and rate-limiting enzyme in the steroidogenic pathway, converting cholesterol to pregnenolone, thereby impacting steroid hormone production, including glucocorticoid production [9]. The *CYP11A1* gene encodes a member of the cytochrome P450 superfamily of enzymes and is primarily expressed in the adrenal cortex. In addition, testis, ovary, placenta, thymus, and intestine also express CYP11A1 [9, 10]. The *CYP11A1* gene locus on human chromosome 15q23-q24 consists of nine exons and a number of transcription factors regulate *CYP11A1* gene expression. Steroidogenic Factor-1, Activator Protein 2, and several tissue-specific GATA family proteins enhance the transcription of *CYP11A1* through binding to the *CYP11A1* promoter site [11–17]. The *CYP11A1* promoter region contains a number of binding sites for the vitamin D receptor, the nuclear hormone receptor for vitamin D3, and vitamin D3 regulates *Cyp11a1* expression [15]. CYP11A1 drives an alternative pathway of vitamin D metabolism and activation, converting it to 20-hydroxyvitamin D3 and other active metabolites [18].

In the present pilot study, we determined the levels of CYP11A1 in PA children and identified, for the first time, that in activated peripheral blood CD4^+^ T cells from PA children compared to healthy controls, the gene and protein levels were significantly increased. *CYP11A1* mRNA levels correlated with CD4^+^ T cell IL-13 production and to outcomes of oral food challenge. Prevention of CYP11A1 enzymatic activity by the inhibitor aminoglutethimide (AMG) or attenuation of *CYP11A1* gene expression using a *CYP11A1* CRISPR/Cas9 KO plasmid suppressed the production of IL-13.

## Results

### Subject characteristics

Thirty-three PA subjects (physician diagnosed or a history of a reaction to peanut) were enrolled and completed the study. Among the PA children, 24 were male and 9 were female, with ages ranging from 3–20 years (median, 8 years). PA children had a median peanut-specific IgE (sIgE) level of 2.77 kU_A_/L (range ≤0.1->10); median sIgE to Ara h 2 of 0.79 kU_A_/L (range ≤0.1->100); median total IgE level of 525 kU/L (range 23.5–4068); and a median skin prick test to peanut of 13.5 mm (range 3–28.5 mm). None of the subjects had both a negative skin prick test to peanut and undetectable levels of sIgE to peanut. Double blind, placebo controlled oral food challenge (DBPCOFC) to peanut resulted in 19 patients (58%) who failed and 14 (42%) who continued to open challenge. The 11 healthy non-allergic children, 2 males and 9 females, ages 2 to 20 years had undetectable levels of peanut sIgE and sIgE to Ara h 2, non-elevated total IgE levels, and negative skin prick test to peanut. Demographic and clinical features of the study population are shown in Tables 1 and 2.

**Table 1:** Patient and control characteristics.

	33 PA	11 Controls
	Median (range)	Median (range)
Age at screening (year)	8 (3–20)	15 (2–20)
Males/Females	24/9	2/9
SPT to peanut (mm)	13.5 (3–28.5)	0
sIgE to Ara h2 (KU_A_/L)	0.79 (0.05–101)	Undetectable
sIgE to peanut CAP (KU_A_/L)	2.77 (0.175–101)	Undetectable
Total IgE (KU/L)	525 (23.5–4068)	All <25

**Table 2:** Patient characteristics and results of DBPCOFC.

Number	Age (y)	Sex	OFC to PE	SPT to PE (mm)	sIgE to PE kUA/L	sIgE Ara h2 kUA/L	Total IgE KU/L
1	7	Male	(+)	12	9.26	5.86	638
2	10	Female	(-)	5.5	2.53	0.05	919
3	6	Female	(-)	3	0.175	0.05	42
4	4	Male	(-)	10.5	0.175	0.15	63
5	10	Male	(-)	8.5	1.2	1.47	1400
6	5	Male	(+)	22	5.96	2.82	306
7	7	Female	(+)	15	1.25	1.68	227
8	12	Female	(+)	7	41.5	0.05	1100
9	6	Male	(-)	15.5	2.98	1.74	594
10	5	Male	(-)	15	3.57	0.41	990
11	11	Male	(+)	23	2.32	0.5	612
12	11	Female	(+)	16	101	71.3	4068
13	6	Male	(+)	12	3.35	4.35	96
14	11	Male	(-)	9.5	4.78	0.77	879
15	10	Male	(-)	5.5	1.44	0.05	45
16	8	Male	(+)	13.5	2.39	1.3	236
17	10	Female	(+)	14	2.06	0.54	152
18	13	Male	(-)	10.5	0.175	0.05	56
19	3	Male	(+)	15.5	48.1	31.9	161
20	5	Female	(-)	11	0.175	0.18	398
21	9	Male	(+)	14.5	1.26	0.82	180
22	5	Female	(-)	8	0.86	0.05	425
23	13	Male	(+)	28.5	8.82	6.18	943
24	11	Male	(-)	8	0.27	0.21	525
25	5	Male	(+)	25	101	101	1709
26	20	Male	(+)	22.5	3.68	0.79	23.5
27	6	Male	(-)	8	4.86	0.05	670
28	8	Female	(+)	17	21.2	22.1	1722
29	7	Male	(+)	10	2.77	2.31	940
30	8	Male	(+)	27	0.39	0.39	106
31	13	Male	(+)	21	4.22	4.22	1460
32	17	Male	(-)	4.5	1.7	0.3	2197
33	4	Male	(+)	19.5	4.31	3.27	227

### CYP11A1 mRNA and protein levels are increased in activated peripheral blood CD4^+^ T cells from PA children

**Fig 1:**
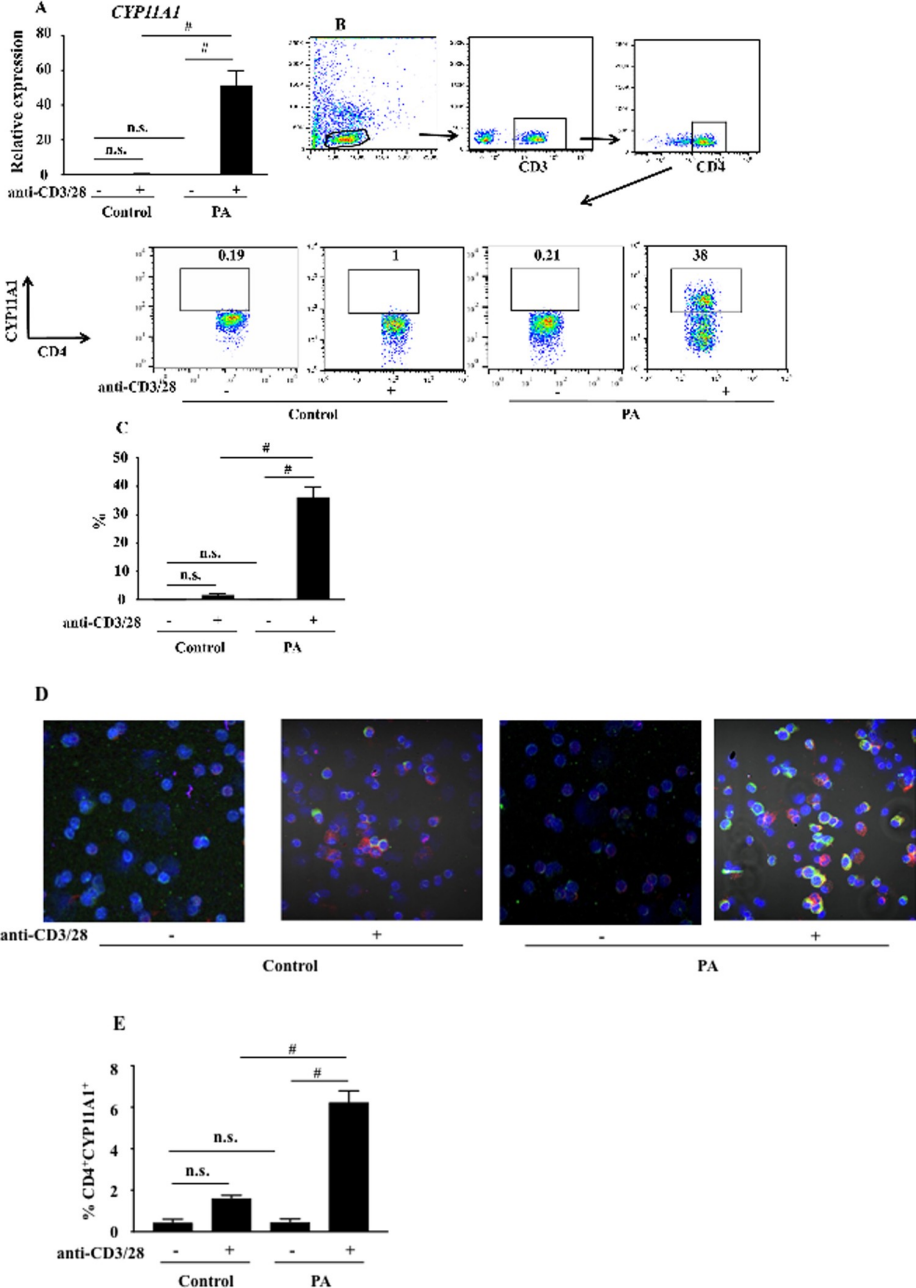
CYP11A1 is expressed in activated and non-activated CD4^+^ T cells from PA children. PBMCs from PA children and healthy controls were activated with anti-human CD3/CD28 for 48 hours. (A) *CYP11A1* mRNA expression detected by qPCR in PBMCs from PA children (n = 33) and controls (n = 11). (B) Representative flow- cytometric analysis of CYP11A1 expression in CD4^***+***^ T cells. (C) Percentages of CYP11A1^***+***^CD4^***+***^T cells (PA children (n = 18) and controls (n = 11). (D) Representative immunocytochemistry staining for CYP11A1. CYP11A1 (green), CD4^***+***^ T cells (red), cell nuclei (DAPI, blue), and coincidence of CYP11A1^***+***^ and CD4^***+***^ T cell staining (yellow). (magnification x200). (E) Percentages of CYP11A1^***+***^CD4^***+***^T cells detected by immunocytochemistry (PA children (n = 18) and controls (n = 11). Data are expressed as means±SEM. #P<0.001, n.s.: not significant.

We examined the expression of *CYP11A1* mRNA in peripheral blood mononuclear cells (PBMCs) from PA children. Following incubation of PBMCs with antibodies targeting T cells (anti-CD3/CD28), *CYP11A1* mRNA expression was increased approximately 50-fold in cells from PA children compared to controls (Fig 1A, P<0.001); no increases were seen in non-activated cells. Using flow cytometry to directly identify CD4^+^CYP11A1^+^ T cells, the percentage of CYP11A1^+^ cells in the total CD4^+^ T cell population (36.3±3.52%) was significantly increased in PA children compared to the non-allergic controls (1.7±0.51%) (Fig 1B and 1C P<0.001); few, if any non-activated cells expressed CYP11A1 and similarly, few, if any, non-CD4^+^ T cells expressed CYP11A1.

Immunocytochemical staining of PBMCs with an antibody specific for CYP11A1 protein demonstrated that the protein was localized to the cell membrane, cytosol and nucleus (Fig 1D). When examined directly, there were few CD4 and CYP11A1 double-positive cells in peripheral blood cells from healthy controls whereas the number of these double-positive cells was significantly increased in peripheral blood cells from PA children following activation with anti-CD3/anti-CD28 (Figs 1D and S1, P<0.001); no increases were seen in non-activated cells from either group. As shown in Fig 1D, CYP11A1 and CD4 were co-localized in the same cells from PA children (and the few in healthy controls).

### IL-13 mRNA and protein expression are increased in PA children

**Fig 2:**
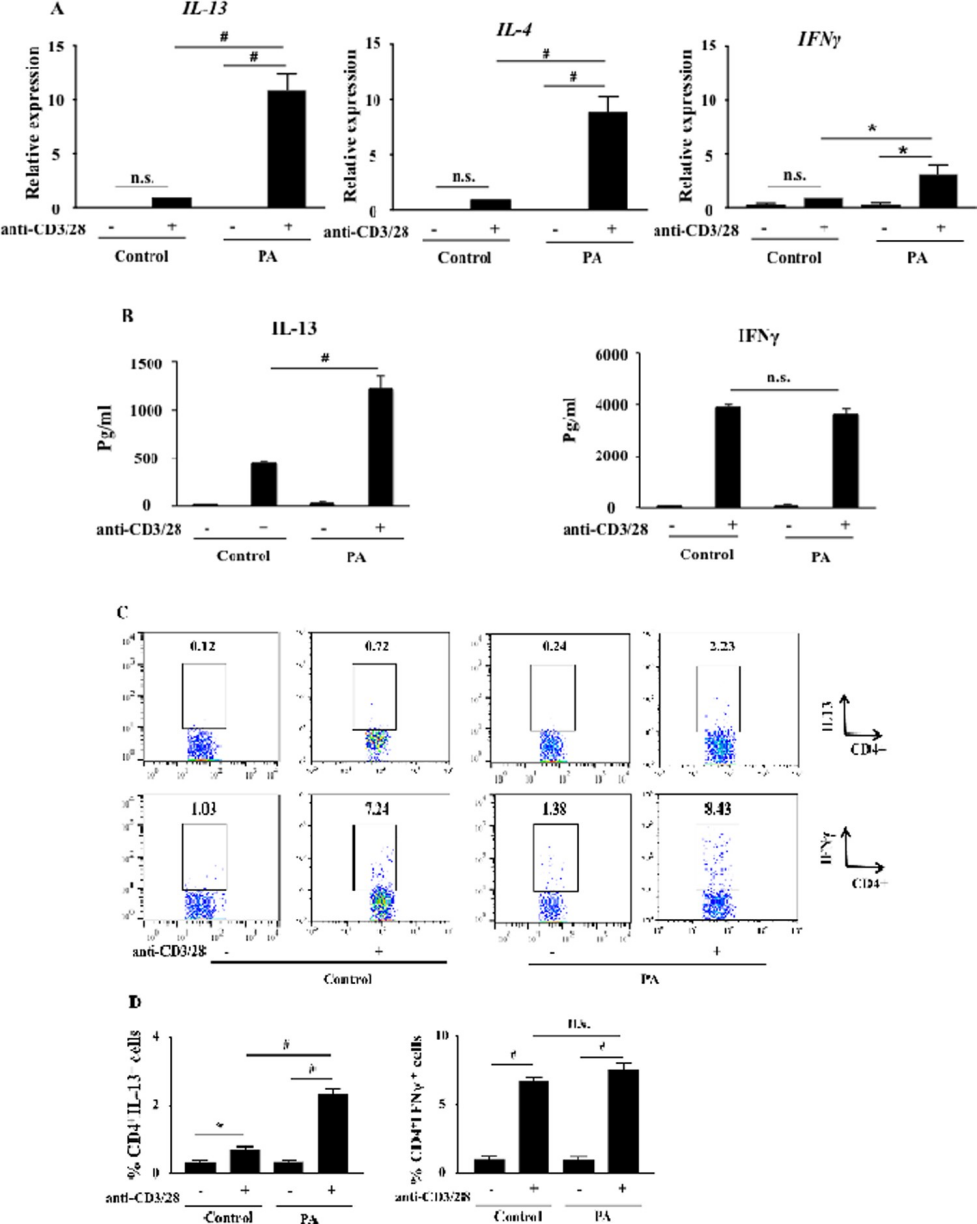
Th2 and Th1 cytokine mRNA and protein expression. (A) *IL13*, *IL4* and *IFNγ* mRNA expression levels were monitored by qPCR (PA children (n = 33) and controls (n = 11)). (B) IL-13 and IFNγ production were measured by ELISA (PA children (n = 33) and controls (n = 11)). (C) Representative flow cytometric analysis of IL-13 and IFNγ expression in activated and non-activated CD4^+^ T cells. (D) Percentages of CD4^+^IL-13^+^T cells and CD4^+^IFNγ^+^T cells (PA children (n = 18) and controls (n = 11)). Data are expressed as means±SEM. *P<0.05, #P<0.001, n.s.: not significant.

The type 2 cytokines, IL-4 and IL-13, play critical roles in IgE-mediated intestinal peanut allergy in humans and mice [8, 19]. Following activation with anti-CD3/anti-CD28, PBMCs from PA children expressed significantly higher levels of *IL13* and *IL4* mRNA (Fig 2A, P<0.001) and produced significantly more IL-13 than the controls (Fig 2B, P<0.001); *IFNγ* mRNA levels were increased to a lesser degree and IFNγ production was the same in both groups; no increase in cytokine production was seen in non-activated cells. The percentage of CD4^+^IL-13^+^ cells detected by flow cytometry was also significantly increased in PA children compared to the controls following activation with anti-CD3/anti-CD28 (Fig 2C and 2D, P<0.001). The percentage of CD4^+^IFNγ^+^ cells was the same in both groups. The very low percentage of CD4^+^IL-13^+^ and CD4^+^IFNγ^+^ cells was the same in both groups in the absence of activation with anti-CD3/anti-CD28.

### Levels of *CYP11A1* mRNA are correlated to levels of IL-13 and sIgE to Ara h 2

**Fig 3:**
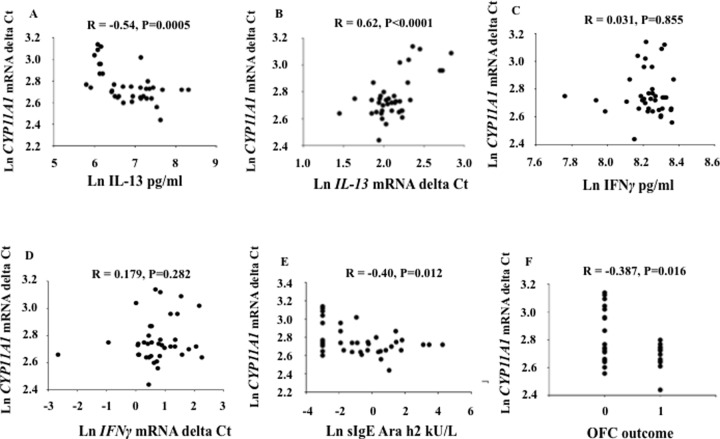
Correlations between *CYP11A1* mRNA, IL-13 and clinical outcomes in PA children (n = 33) and healthy controls n = 11). lower delta Ct values correspond to higher mRNA levels. (A) Significant correlation between *CYP11A1* mRNA expression and IL-13 levels (P<0.001, lower Ct values mean higher mRNA levels). (B) Significant correlation between *CYP11A1* mRNA expression and *IL13* mRNA expression (P<0.001). (C) No significant correlation between *CYP11A1* mRNA expression and IFNγ levels (P>0.05). (D) No significant correlation between *CYP11A1* mRNA expression and *IFNγ* mRNA expression (P>0.05). (E) Significant correlation between *CYP11A1* mRNA and serum levels of sIgE to Ara h 2 (P<0.05). (F) Significant correlation between *CYP11A1* mRNA and outcomes of oral food challenge (1 = positive, 0 = negative (no reaction) (P<0.05). Analyses were performed using Pearson correlation. The data were expressed as log-transformed data.

Since *CYP11A1* mRNA was highly expressed in activated CD4^+^ T cells from PA children, we determined the correlations between *CYP11A1* mRNA expression levels and IL-13 in the cohort. Despite the modest number of patients, there were significant correlations between *CYP11A1* mRNA levels and levels of IL-13 production (Fig 3A, r = -0.54, P<0.001) as well as to *IL13* mRNA expression levels (Fig 3B, r = 0.62, P<0.001). In contrast, no correlations were detected between *CYP11A1* mRNA expression and *IFNγ* production or IFNγ mRNA levels (Fig 3C and 3D, r = 0.031, P>0.05; r = 0.179, P>0.05; respectively). In addition, levels of *CYP11A1* mRNA expression were correlated to levels of sIgE to Ara h 2 (Fig 3E, r = -0.40, P<0.05).

### Levels of *CYP11A1* mRNA are correlated to outcomes of DBPCOFC

Oral challenge to peanut was carried out in all PA patients regardless of history or evidence of sensitization by skin test or sIgE results. Despite the relatively small numbers of responders (58%) and non-responders (42%), we determined if the levels of *CYP11A1* mRNA expression were correlated to outcomes of DBPCOFC. Using a binary assessment of outcomes in DBPCOFC (1 = positive, 0 = negative (no reaction)), levels of *CYP11A1* mRNA expression were correlated to outcomes of DBPCOFC (Fig 3F, r = -0.387, P<0.05).

### Inhibition of CYP11A1 suppresses IL-13 production

**Fig 4:**
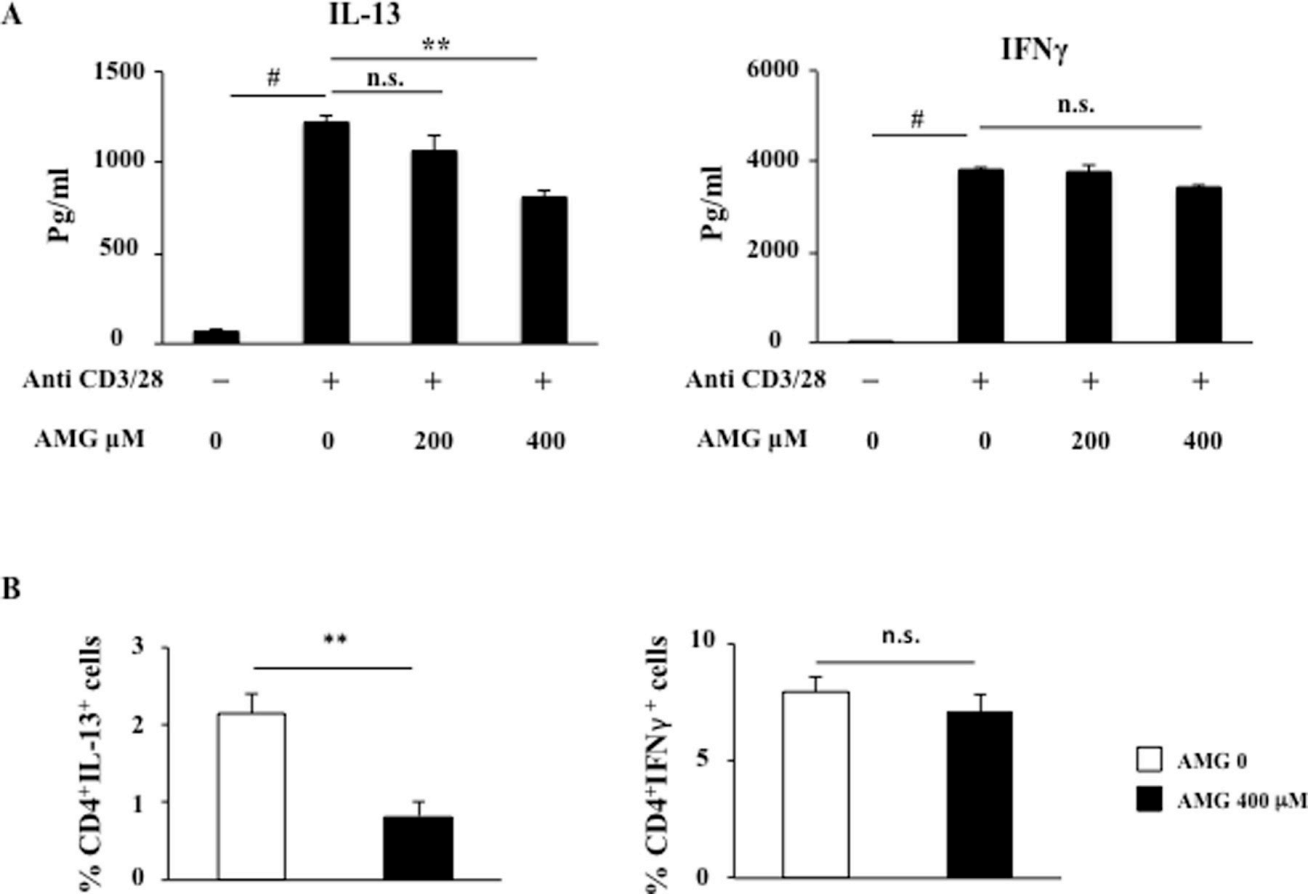
Effects of CYP11A1 inhibition on cytokine production. (A) IL-13 and IFNγ production were measured in culture supernatant of activated, patient PBMCs treated with aminoglutethimide or vehicle by ELISA (n = 12). (B) Percentages of CD4^+^IL-13^+^ cells and CD4^+^IFNγ^+^ cells (n = 12). **P<0.01, #P<0.001, n.s.: not significant.

To establish the role of CYP11A1 in IL-13 production in human CD4^+^ T cells, as shown in the mouse, we determined the effect of a specific inhibitor of enzymatic CYP11A1 activity, aminoglutethimide. PBMCs from PA children were activated with anti-CD3/anti-CD28 in the presence or absence of aminoglutethimide for 48 hours. Addition of aminoglutethimide (400 μM) resulted in significantly decreased levels of IL-13 in the culture supernatant (Fig 4A). Levels of IFNγ were not affected by the inhibitor confirming the absence of toxicity of the drug in concert with no loss of cell viability (S1 Fig in the Online Supplement). In parallel, the percentage of CD4^+^IL-13^+^ cells was also significantly decreased by aminoglutethimide (400 μM) from 2.15±0.25% to 0.83±0.19%. P<0.01 (Fig 4B). The percentage of CD4^+^IFNγ^+^ cells was not significantly reduced by aminoglutethimide at 400 μM (7.95±0.66% to 7.10±0.72%. P>0.05). As baseline levels of CYP11A1 expression were barely detectable and no increases in CYP11A1 expression were seen in cells from healthy controls activated with anti-CD3/anti-CD28, studies with the inhibitor in the control cells were not carried out.

### CYP11A1 CRISPR/Cas9 KO reduces Th2 cytokine production in SUP-T1 cells *in vitro*

**Fig 5:**
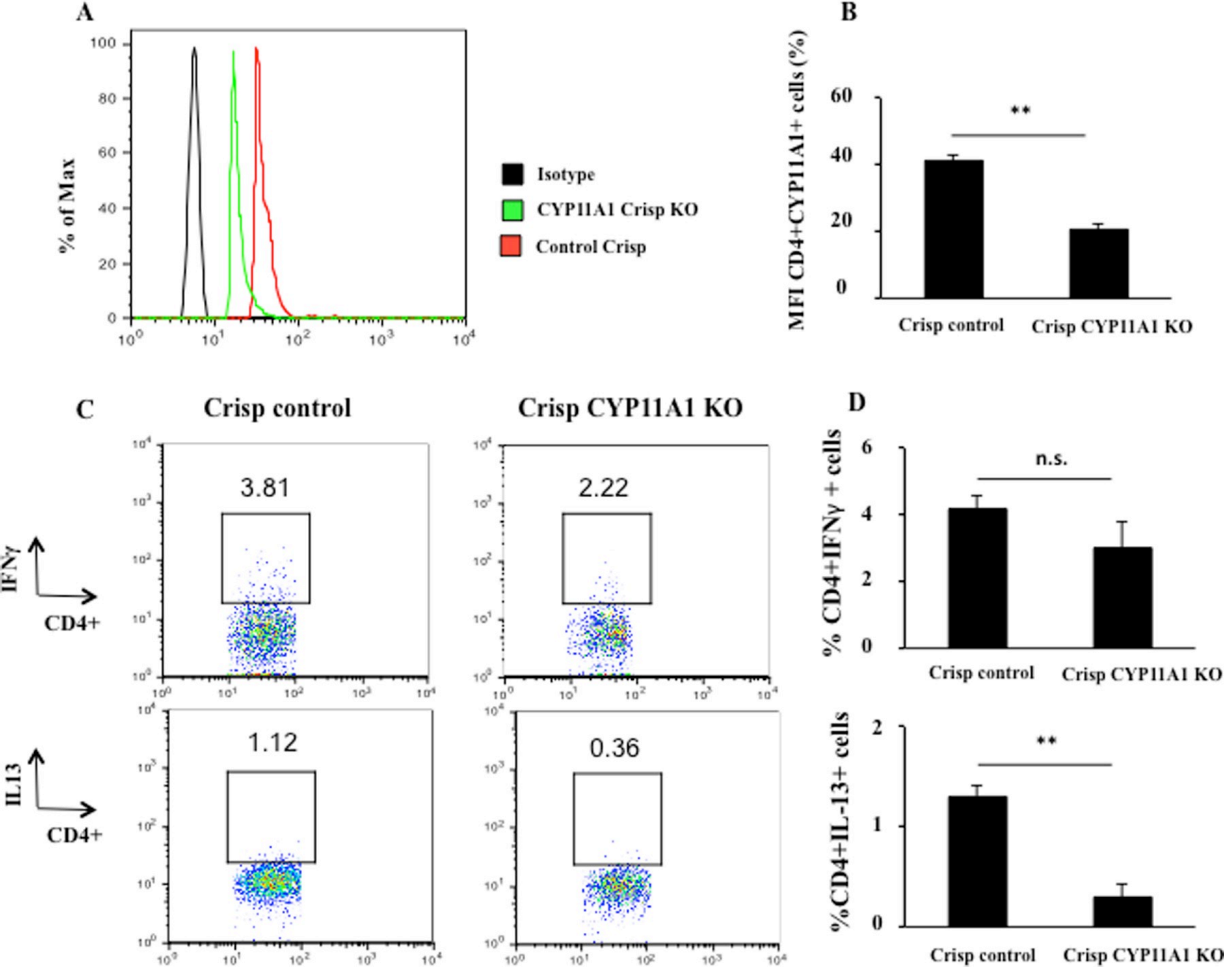
*CYP11A1* CRISPR/Cas9 KO inhibits Th2 cytokine production in SUP-T1 cells. (*A*) Representative flow-cytometric analysis of CYP11A1 expression after transfection with *CYP11A1* CRISPR/Cas9 KO plasmid or scrambled control CRISPR/Cas9 plasmid. (*B*) Quantitative analysis of double CYP11A-positive and CD4-positive cells. (*C*) Representative flow cytometric analysis of cytokine expression in CD4^+^ T cells after transfection. (*D*) Percentages of CD4^+^IL-13^+^ cells and CD4^+^IFNγ^+^ cells. Data are from two independent experiments. **P < 0.01, n.s.: not significant, compared with scramble CRISPR/Cas9 plasmid group.

In addition to targeting the enzymatic activity of CYP11A1, to further confirm the role of CYP11A1 in IL-13 production in human cells, SUP-T1 cells (human T cell lymphoblastic lymphoma derived cells) were transfected with a control (scrambled) or *CYP11A1* CRISPR/Cas9 KO plasmid. SUP-T1 cells were chosen since they highly express CD4 and are a suitable transfection host. Forty-eight hours after transfection, the cells were stimulated with PMA and ionomycin in the presence of brefeldin A for 4 hours. Flow cytometric analysis for GFP indicated that there were 18.3±1.2% and 21.6±2.4% GFP-positive cells following transfection of *CYP11A1* CRISPR/Cas9 KO plasmid or scrambled control CRISPR/Cas9 plasmid, respectively. Among the GFP-positive cells, 41.1±2.1% of cells receiving the scrambled CRISPR/Cas9 plasmid were double-positive for CYP11A1 and CD4. This number was significantly reduced to 20.5±1.8% in cells transfected with the *CYP11A1* CRISPR/Cas9 KO plasmid (Fig 5A and 5B, P<0.01). After transfection of the *CYP11A1* CRISPR/Cas9 KO plasmid, the percentage of CD4^+^IL-13^+^ cells decreased to 0.3±0.13% compared with 1.3±0.11% in cells transfected with the scrambled CRISPR/Cas9 plasmid; in parallel, the percentage of CD4^+^ IFNγ^+^ cells did not significantly change after transfection of *CYP11A1* CRISPR/Cas9 KO plasmid or scrambled control CRISPR/Cas9 plasmid (Fig 5C and 5D, P<0.01, P>0.05 respectively).

## Discussion

Gene expression studies have provided insight into complex molecular mechanisms leading to the development of allergic diseases. We previously found that *Cyp11a1* mRNA expression was increased 3-fold in the jejunal homogenates from peanut-sensitized mice [8] and *Cyp11a1* mRNA expression was approximately 300-fold higher in polarized Th2 cells compared to Th1 cells *in vitro* [8]. Silencing of *Cyp11a1* by shRNA reduced Th2 cytokine (*IL4* and *IL13)* mRNA and protein expression in polarized Th2 cells [8]. The steroidogenic potential of activated CD4^+^ Th2 cells, demonstrated by production of pregnenolone in a CYP11A1-dependent manner, and in association with type 2 cytokine expression has been demonstrated in different mouse systems [8, 20, 21] and identified CYP11A1 as playing a key role in the regulation of T-cell mediated allergic responses. Translating these findings from an experimental model of peanut-induced intestinal anaphylaxis in mice to PA children, we now demonstrate, for the first time, that *CYP11A1* mRNA and protein expression were significantly increased in activated, but not in non-activated, peripheral blood CD4^+^ T cells from PA children compared to controls and expression levels of *CYP11A1* mRNA were correlated to levels of IL-13.

Presently, the diagnosis of food allergy is based mainly on patient history, skin prick tests, and/or serum allergen-specific IgE levels to food proteins or a component. However, sIgE levels and skin prick tests indicate allergen sensitization rather than clinically-significant food allergy and they do not correlate well with the outcome of DBPCOFC [22]. Moreover, both tests are associated with a high rate of false positives and are often the basis for inappropriate and demanding food elimination [5]. With the costly and somewhat risky DBPCOFC remaining as the current gold standard for determining clinical peanut allergy, development of predictors of peanut allergy not simply peanut sensitization represents an important unmet need.

In the current study, in children with evidence of sensitization to peanut (positive skin prick test) and/or peanut-specific IgE) and allergy confirmed in many on DBPCOFC, we determined if expression of CYP11A1 was increased in peripheral blood CD4^+^ T cells from PA children. In the PA children, CYP11A1 was highly expressed in activated peripheral blood CD4^+^ T cells compared to controls. In activated T cells (anti-CD3/anti-CD28) from PA children, *CYP11A1* mRNA expression was increased approximately 50-fold compared to controls. In parallel, the percentage of CD4^+^CYP11A1^+^ cells was significantly increased in the PA children compared to the non-allergic children; few non-CD4^+^ T cells expressed CYP11A1. *CYP11A1* mRNA and protein expression levels were undetectable or very low in non-activated peripheral blood CD4^+^ T cells from both PA children and controls.

In both humans and mice, the type 2 cytokines, IL-4 and IL-13, play critical roles in the development of IgE-mediated intestinal peanut allergy [8, 19]. The percentage of CD4^+^IL-13^+^ cells was significantly increased in the peripheral blood of PA children. Following activation with anti-CD3/anti-CD28, cells from PA children expressed significantly higher levels of *IL13* and *IL4* mRNA and produced significantly more IL-13 following culture. *IFNγ* mRNA levels were increased to a significantly lesser degree and IFNγ production was not different between the PA children and controls. The percentages of CD4^+^IFNγ^+^ cells were the same in both groups. As with the increases in CYP11A1, in the absence of activation with anti-CD3/anti-CD28, levels of *IL13*, *IL4*, and *IFNγ* mRNA were undetectable or very low and no increase in IL-13 and IFNγ production was seen in the PA children and controls.

In mice, peanut-induced intestinal allergy was shown to be mediated through a mast cell-IgE-FcεR1-IL-13 pathway [23]. *In vitro*, allergen-stimulated T cells and T-cell clones generated from patients with peanut allergy produced increased levels of the type 2 cytokines IL-4, IL-5, and IL-13 [24]. In the present study, there were significant correlations in the PA patients between *CYP11A1* mRNA levels to levels of *IL13* mRNA expression and IL-13 production in activated CD4^+^ T cells, as well as to serum levels of sIgE to Ara h 2. In contrast, no correlations were detected between levels of *CYP11A1* mRNA expression to *IFNγ* mRNA or IFNγ production. Surprisingly, although the numbers of responders and non-responders to oral peanut challenge were small, we determined if the levels of *CYP11A1* mRNA, in parallel to levels of IL-13 production and sIgE to Ara h 2, were correlated to outcomes of DBPCOFC to peanut. Although modest, a positive correlation was seen. Confirmation in a larger cohort of patients is now needed to determine if levels of *CYP11A1* mRNA in activated CD4^+^ T cells can serve as a predictor of peanut allergy.

To establish the role of CYP11A1 in IL-13 production in human CD4^+^ T cells as shown in the mouse [8], two approaches were taken. We previously showed that the enzymatic activity of CYP11A1, resulting in the conversion of cholesterol to pregnenolone, could be blocked by aminoglutethimide [8]. Inclusion of aminoglutethimide in the cultures of PA patient cells significantly decreased the levels of IL-13 production in the culture supernatant. This was not due to drug toxicity or effects on cell viability as IFNγ production was not affected by the inhibitor. The percentages of CD4^+^IL-13^+^ cells were also significantly decreased by aminoglutethimide. To further confirm the relationship between CYP11A1 and IL-13, we used genomic editing with CRISPR/Cas9 to achieve knockdown of the *CYP11A1* gene. We transfected the human T cell line, SUP-T1, with control (scrambled) or the *CYP11A1* CRISPR/Cas9 KO plasmid. Among the cells transfected with the CRISPR/Cas9 KO plasmid, the number of CD4^+^ T cells expressing CYP11A1 was significantly reduced by more than 50%, as were the number of IL-13^+^ CD4^+^ T cells.

Although these results implicate a role for CYP11A1 in the development of peanut allergy, a number of important questions arise. The cohort was chosen, as best as possible, to eliminate patients with active atopic dermatitis or asthma. Although the results separated PA children from healthy controls, it is currently unclear if the findings are specific to PA children *per se*; the findings may reflect a more general response in allergic diseases where CYP11A1 and IL-13 play a central role and where CYP11A1 is a major regulator of IL-13 production. Of note here is the (negative) regulation of CYP11A1 expression by vitamin D3 [15] and the role of vitamin D3 in attenuating certain allergic manifestations, albeit controversial [25]. Further studies are now required with larger numbers and in patients with diverse allergic disorders to determine if similar correlations between CYP11A1 and IL-13 levels are present and whether there may be disease-specific associations. Increased numbers of PA patients would also provide broader ranges of CYP11A1 and IL-13 expression levels to more accurately determine and validate predictive cut-off levels of these markers. This may become even more predictive if peanut specific CD4^+^ T cells rather than polyclonal T cell activation are studied, which was not possible here. Even considering these caveats, the results identify a CYP11A1-IL-13 axis in the development of peanut allergy in children, supporting potential targeting of CYP11A1 as a novel treatment strategy in controlling peanut allergy. Future studies will determine the validity of these possibilities. This study confirms, as in mice [8, 20], that CYP11A1 is a major regulator of IL-13 production. Little is currently known about the molecular mechanisms mediated by activity of this enzyme that results in IL-13 production. Transcription and enzymatic activity of CYP11A1 is known to be susceptible to vitamin D which primarily transmits a signal through the transcription factor vitamin D receptor, a transcriptional repressor of CYP11A1 [15]. As a key regulator of steroidogenesis, CYP11A1 enzymatic activity is the first and rate-limiting step in the biosynthesis of all steroid hormones. Activation of CYP11A1results in a spectrum of steroid hormones, including glucocorticoids that are known to play a role in T-cell function [13, 26]. T cells express androgen receptor and estrogen receptor which impact cytokine gene transcription. T cells express many of the steroid metabolic enzymes [27]. It is possible that one of the metabolites of the steroidogenesis pathway, in concert with T-cell receptor activation, regulates IL-13 transcription factor expression and IL-13 production.

## Materials and methods

### Study population

PA (n = 33) and healthy control subjects (n = 11) ages 3–20 years were recruited from the Pediatric Allergy clinics at National Jewish Health and surrounding community. PA subjects were defined as having a physician-diagnosed peanut allergy or a history of a reaction to peanut; evidence of sensitization to peanut was determined by skin prick and blood testing. Sensitization was defined as a mean skin prick test 3 mm greater than the negative control [28], or sIgE to peanut >0.35 kU_A_/L [29]. The study was approved by the National Jewish Health Institutional Review Board. Written informed consent and assent were obtained from all subjects and/or their parents as age-appropriate. Exclusion criteria included severe and uncontrolled asthma or atopic dermatitis, life-threatening anaphylaxis to peanut within the prior year, and active eosinophilic gastrointestinal disease. The healthy non-allergic children had no history of food sensitivity or other allergic diseases, had never reacted to peanut ingestion and were currently consuming peanut in their diets. All PA children underwent clinical evaluation, skin prick test, total IgE, peanut-specific IgE, IgE to peanut components, and DBPCOFC. Peripheral blood was drawn in heparin vacutainer tubes (BD, Franklin Lakes, NJ) from all children for isolation of PBMCs.

### Skin prick test

Skin prick tests were performed on the back using positive control (histamine), negative control (saline), and commercial peanut extract (Stallergenes Greer, Cambridge, MA) following published guidelines [30].

### Peanut-specific IgE and total IgE

Serum levels of total IgE, peanut sIgE and sIgE to Ara h 2 were measured using ImmunoCAP (Thermo Fisher Scientific, Waltham, MA) according to the manufacturer's instructions in a CLIA-approved laboratory.

### Double-blind placebo controlled oral food challenge

DBPCOFCs were performed by a nurse practitioner and/or physician with expertise in food allergy and conducting oral food challenges. The DBPCOFC consisted of randomized ingestion of placebo (oat flour) on one day and ingestion of peanut flour in vehicle on the second day. Vehicles included apple sauce or chocolate pudding, per patient preference. Doses were given in 13 increasing doses, every 15 minutes up to a cumulative dose of 5 grams of peanut protein. Objective symptoms led to stopping the oral food challenge, and were treated per provider discretion. Subjective symptoms were assessed appropriately, and the challenge proceeded with delayed, repeated or continued dosing. If both days were passed, the subject was then given an open serving of peanut butter, containing 7 grams of peanut protein.

### T cell cultures

PBMCs were isolated from peripheral venous blood following Ficoll-Hypaque (GE Healthcare, Uppsala, Sweden) density gradient centrifugation and activated with anti-human CD3/anti-CD28 (2 μg/ml) monoclonal antibodies (eBioscience, San Diego, CA) for 48 hours. Cultured cells were collected for RNA isolation, flow cytometry and immunocytochemistry analyses. Cell supernatants were collected and assayed for cytokine production.

### Quantitative real-time PCR

RNA was isolated from cultured cells using Trizol (Invitrogen, Carlsbad, CA). cDNA was generated with the iScript cDNA synthesis kit (Bio-Rad Laboratories, Hercules, CA). Quantitative real-time PCR was performed on the ABI Prism 7500 sequence detection system (Applied Biosystems, Foster City, CA). All primers and probes used were purchased as TaqMan Gene Expression Assays from Applied Biosystems. Human GAPDH was used as a reference [31, 32]. Fold changes were calculated by using the delta-delta cycle threshold (ΔΔC_T_) method.

### Flow cytometry and immunocytochemistry

Intracellular cytokine and CYP11A1 staining was performed as described previously [33]. The cells were labeled with anti-human CD3 and CD4 antibodies (eBioscience) and stained for intracytoplasmic CYP11A1 (Abcam, Cambridge, MA), IL-13, and IFNγ (eBioscience) using an intracellular staining kit (eBiosciences) according to the manufacturer’s protocol. Cells were analyzed on an LSR II (BD Biosciences, San Jose, CA) using the FlowJo software (Tree Star, Ashland, OR). Slides were prepared from cultured PBMCs using cytospin. Numbers of cultured cells expressing CD4 and CYP11A were identified by ICC staining using anti-human CD4 (eBiosciences) and anti-CYP11A1 (Abcam) and analyzed using an LSM 700 confocal microscope (Carl Zeiss, Thornwood, NY). Quantitative analysis was performed by counting CYP11A1^+^ and CD4^+^ cells under the LSM 700 confocal microscope.

### Cytokine production

Levels of IL-13 and IFNγ in cell culture supernatants were measured by ELISA (eBioscience) as described by the manufacturer.

### Inhibition of enzymatic CYP11A1 activity

Aminoglutethimide (Sigma, St. Louis, MO) was dissolved in 0.1M hydrochloric acid (pH 1.0) and diluted with RPMI medium in *in vitro* studies. The final concentration of hydrochloric acid was less than 0.05%. Aminoglutethimide was added at different doses based on our previous reports [8, 20].

### Human SUP-T1 cell line culture and *CYP11A1* CRISPR/Cas9 KO plasmid

SUP-T1 cells (ATCC, Manassas, VA) were plated and cultured overnight to reach 60 to 80% confluency. Cells were transfected with a human *CYP11A1* CRISPR/Cas9 knockout plasmid with a GFP insert (Santa Cruz Biotechnology, Santa Cruz, CA) or a scrambled control CRISPR/Cas9 plasmid using Xfect transfection reagent (Clontech, Mountain View, CA) according to the manufacturer's instructions. Medium was changed after 4 hours of transfection. Forty-eight hours after transfection, the cells were stimulated with PMA and ionomycin in the presence of brefeldin A (an inhibitor of intracellular protein transport). Intracellular staining and flow cytometric analysis were as described above. Cell viability assessed by trypan blue dye exclusion remained at 90% following transfection.

### Statistical analyses

One-way ANOVA was used to compare mean levels between the four groups: PA group with and without activation; control group with and without activation. Comparisons for all pairs utilized the Tukey-Kramer Honest Significant Difference test. Pearson correlations were computed between CYP11A1 and IL-13, outcomes of DBPCOFC, and sIgE to Ara h 2 after transforming all of these variables to the natural log scale. DBPCOFC was binary (1 = positive, 0 = negative (no reaction)), and hence correlations between this and other continuous variables are referred to as point-biserial correlations. P values for significance were set at 0.05 for all tests (two-tailed, unpaired). Untransformed data were summarized using mean±SEM as indicated in the text.

## Supporting information

S1 Fig Cell viability in PBMCs from PA children treated with the inhibitor aminoglutethimide (n = 12). Data are expressed as means±SEM. n.s., not significant.
(TIFF)
