# Supplementary figures and images for: Expression and activation of the steroidogenic enzyme CYP11A1 is associated with IL-13 production in T cells from peanut allergic children
Source: PLoS One. 2020 Jun 4;15(6):e0233563. doi: 10.1371/journal.pone.0233563 (PMC7272076; doi:10.1371/journal.pone.0233563)

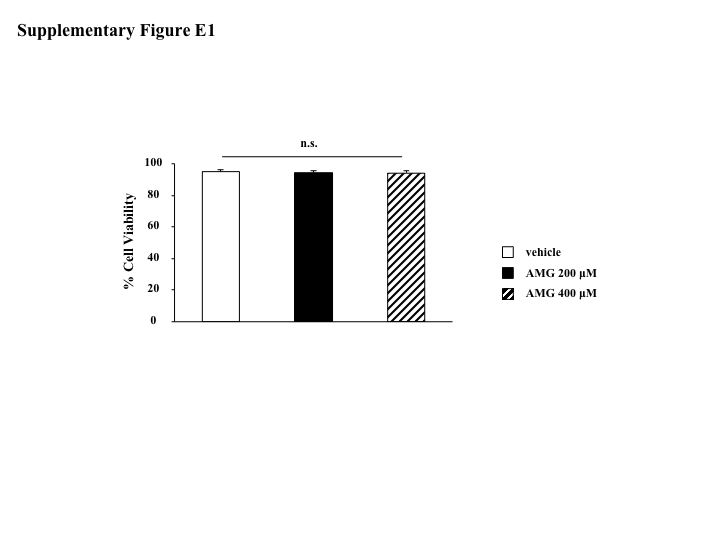

Supplement: S1 Fig — Data are expressed as means±SEM. n.s., not significant. (TIFF) [file pone.0233563.s001.tiff]
